# Analysis of Epileptic Seizures with Complex Network

**DOI:** 10.1155/2014/283146

**Published:** 2014-07-23

**Authors:** Yan Ni, Yinghua Wang, Tao Yu, Xiaoli Li

**Affiliations:** ^1^School of Medical Information Engineering, Jining Medical University, Jining 272067, China; ^2^State Key Laboratory of Cognitive Neuroscience and Learning & IDG/McGovern Institute for Brain Research, Beijing Normal University, Beijing 100875, China; ^3^Center for Collaboration and Innovation in Brain and Learning Sciences, Beijing Normal University, Beijing 100875, China; ^4^Beijing Institute of Functional Neurosurgery, Xuanwu Hospital, Capital Medical University, Beijing 100053, China; ^5^Institute of Electrical Engineering, Yanshan University, Qinhuangdao 066004, China

## Abstract

Epilepsy is a disease of abnormal neural activities involving large area of brain networks. Until now the nature of functional brain network associated with epilepsy is still unclear. Recent researches indicate that the small world or scale-free attributes and the occurrence of highly clustered connection patterns could represent a general organizational principle in the human brain functional network. In this paper, we seek to find whether the small world or scale-free property of brain network is correlated with epilepsy seizure formation. A mass neural model was adopted to generate multiple channel EEG recordings based on regular, small world, random, and scale-free network models. Whether the connection patterns of cortical networks are directly associated with the epileptic seizures was investigated. The results showed that small world and scale-free cortical networks are highly correlated with the occurrence of epileptic seizures. In particular, the property of small world network is more significant during the epileptic seizures.

## 1. Introduction

Epilepsy is a manifestation of abnormal electrical activity in the central nervous system, caused by the imbalance of excitatory and inhibitory synapses [1–3]. To study the generation mechanism of epileptic seizures, the complex network theories have been applied to investigate the structural and functional organizations of underling brain connections [4, 5]. In particular, small-world or scale-free networks are theoretically believed to be associated with rapid information propagation and low wiring cost in the brain [6] and allow coexistence of functional segregation and information integration [7].

To explore the epileptiform behaviors in terms of complex network property in underlying neural networks, three types of neuron models have been introduced: noisy and leaky integrate-and-fire neurons, stochastic Hodgkin-Huxley cells, and Poisson spike-train cell model neurons [4]. Changing parameters such as the synaptic strength, number of synapses per neuron, and proportion of local versus long-distance connections will induce “normal,” “interictal,” and “ictal” epilepsy behaviors. Simulations showed that small world connectivity at the neuronal level plays an important role in the behavior of the networks. For example, Tsodyks found that the coherent activity in randomly connected network with depressing synapses was similar to the bursting [8]. Adding long-distance connections among integrate-and-fire neurons will construct a small world network, which would transit from sustained activity to synchronous bursts of finite duration [9]. Beggs and Plenz replicated their scale-free behavior in a multilayer, feedforward model [10]. They concluded that the most common events are small in spatial scale and short in duration. In addition, networks of oscillatory elements would synchronize when the network contains enough long-distance connections of sufficient synaptic strength [11]. Kötter and Sommer found that small world properties of macaque cortex are associated with the propagation of strychnine-induced epileptiform activity [12].

In this study, to investigate the small world or scale-free network property of functional connectivity in the brain during epileptic seizures, the neural network properties during epileptic seizures were calculated by introducing a multiple mass neural model. Simulated EEG signals were generated under the assumption of small world, scale-free, random, and regular networking, and the relation between simulated EEG signals and the network structure was discussed. The findings supported that the small world and scale-free network can strongly induce the epileptic discharges.

## 2. Methods

### 2.1. Mass Neural Model

**Figure 1:**
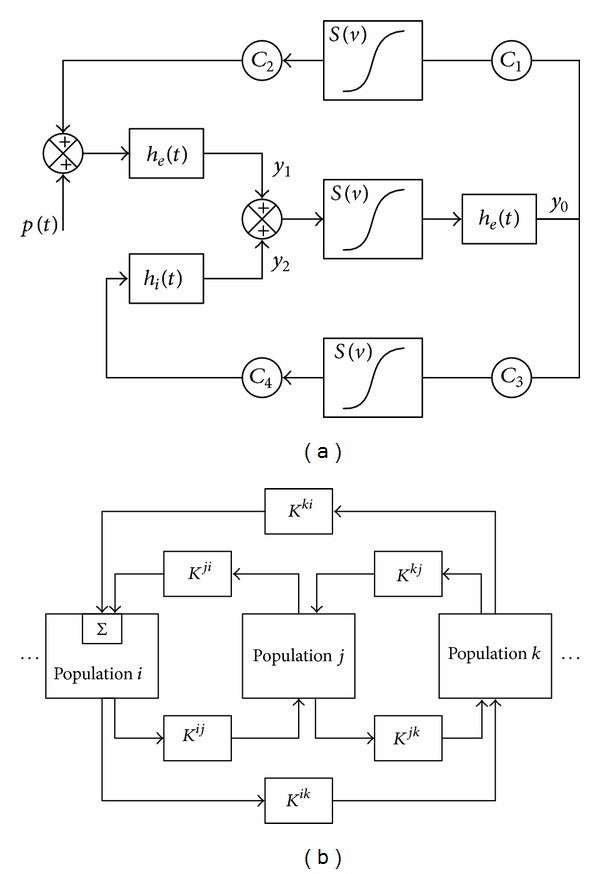
The scheme of multiple mass neural model. (a) One-population mass neural model. (b) The coupled multipopulation neural mass model.

The mass neural model was initially proposed by Lopes da Silva et al. [13] and was later improved and extended by Wending et al. [14, 15]. In the present study we use Wending's model, which is composed of a model of one neural population and a model of multiple coupled neural populations. The model is illustrated in Figure 1.

The population model (Figure 1(a)) contains two interacting subsets: the first is composed of pyramidal cells, which projects to and receives feedback (either excitatory or inhibitory) from the second subset; the second is composed of excitatory neurons and inhibitory neurons, which receives excitatory inputs only [14]. The input *p*(*t*) represents the average density of afferent action potentials. Subset 1 is characterized by two second-order dynamic linear transfer functions, which transfers the average presynaptic pulse density of afferent action potentials (the input) into an average postsynaptic membrane potential (the output). The impulse responses of excitatory and inhibitory neurons are presented by *h*_*e*_(*t*) and *h*_*i*_(*t*), respectively. When the neuron fires, a static nonlinear asymmetric sigmoid function *S*(*v*) would send the average postsynaptic potential to the average pulse density of potentials. There is only one linear transfer function from excitatory neuron in subset 2. Parameters C1–C4 are constants representing average synaptic number. In normal situation, excitatory and inhibitory neurons keep a balance. The change of the ratio between excitatory and inhibitory synaptic gains will trigger epileptic seizure and generate sporadic spikes, rhythmic spikes, and so on.  Figure 1(b) is the multiple coupled neural network model [14], which is composed of a few neural populations. The populations correspond to different brain areas and their interactions are links determined by the parameters *K* ^*ij*^. During the epileptic seizure, the excitatory potentials propagate along axons from one population to another, leading to high voltage, exciting states.

### 2.2. Complex Network

**Figure 2:**
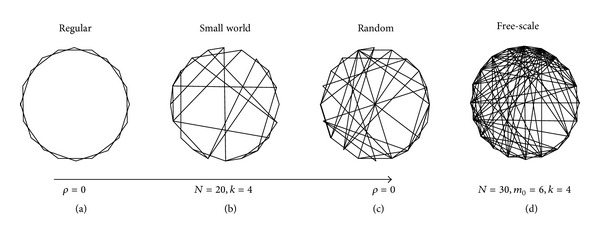
(a)–(c) The random rewiring procedure of the network model from a regular network to a random network without altering the number of nodes or edges. (d) A scale-free network.

Recently, complex network has been drawing attention in fields from physical, biological systems to social constructions [16, 17]. A network is composed of many nodes and links (or edges) linking the nodes. This is mathematically described as a graph. The complexity of a network depends not only on the number of the nodes and links, but also on the interaction dynamics of nodes. Clustering strength among neighboring nodes and their path length are two important indices to indicate the nature of a complex network, in the form of regular network to random network. The network which lies between regular and random networks is called small world network, in which most of the nodes are connected to their nearest neighbors, and a few of nodes are linked over a long range. A typical example is the social network. The manifestation in society follows the “six degrees of separation” concept [18]. The property of small world network was naturally described in [19]. Additionally, a more special network is defined as a scale-free network. Scale-free networks' structure and dynamics are independent of the number of nodes; the connection degree distribution in the scale-free network follows the Yule-Simon distribution (a power-law relationship) [16, 20, 21]. In brief, four typical networks are plotted in Figure 2.

## 3. Results

A coupled mass neural model was applied to explore the relation between the behavior (discharge) and structure of networks. The coupled model has two parameters: the nodes (*n*) and the connectivity (*k*). Here the connectivity denotes the number of links (or edges) a node contacts other nodes in the network. And two tests were conducted. In the first test, the parameters of model are fixed, the simulated EEG discharges (including the epileptic seizures) were obtained by adjusting the connection in the network, introducing regular, small world, free-scale, or random network types, and then the relationship between the epileptic discharges and the structure of network was analyzed. In the second test one of 32 nodes (a mass model) was adjusted to generate a spontaneous epileptic discharge. Then the relationship between the propagation of epileptic discharge and structure of network was investigated.

**Figure 3:**
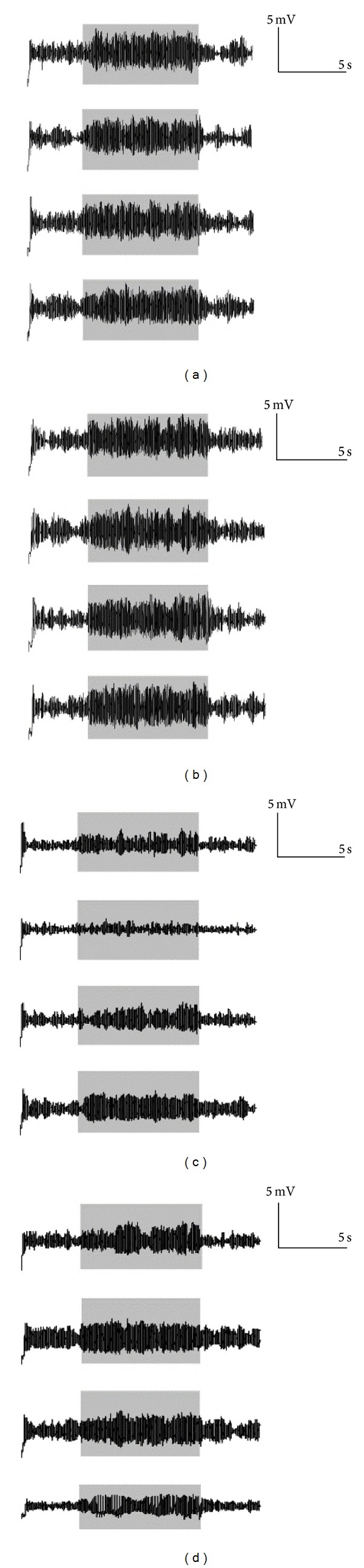
Simulated EEG signals of 32 coupled mass neural models (*n* = 32, *k* = 5). (a) Regular network; (b) small world network; (c) scale-free network; and (d) random network.

Given the network parameters *n* = 32, *k* = 5, the model parameters were set as *A* = 3.55 mV, *B* = 22 mV, *K* ^*ij*^ = 100, and  *i*, *j* = 1,2,…, 32. Other parameters were set according to [22]. The EEG signals in the differently coupled networks are generated, as shown in Figure 3. Only channels 1, 10, 20, and 30 are plotted to save place. Figure 3 shows that the intensity of simulated epileptic discharges was stronger when the coupled network was regular and “small world.”

**Figure 4:**
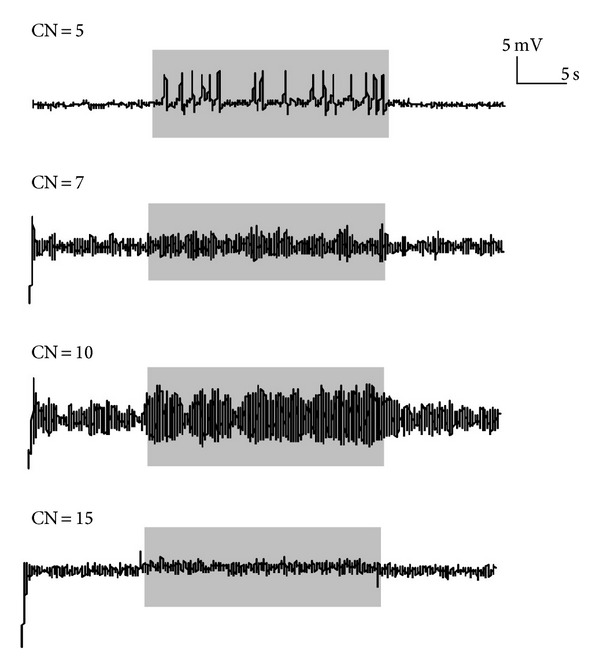
The different epileptic form discharges at the scale-free network (*n* = 32, *k* = 5). CN is the connection number between the node and other nodes.

Furthermore, a scale-free network was studied. The degree distribution in the scale-free network follows a power-law behavior: *p*(*k*) ~ *k* ^−*γ*^, usually 2 ≤ *γ* ≤ 3 [16]. Simulated EEG recordings of a node that exhibit epileptic seizure pattern are shown in Figure 4. CN = 5, 7, 10, and 15 are referred to as the connection number between this node and other nodes. The simulated EEG recordings of this node changed with the CN before seizure (0–5 s). However, the epileptiform discharge at the seizure stage did not follow any principles. This suggests that the scale-free networks have more complicated dynamics.

**Figure 5:**
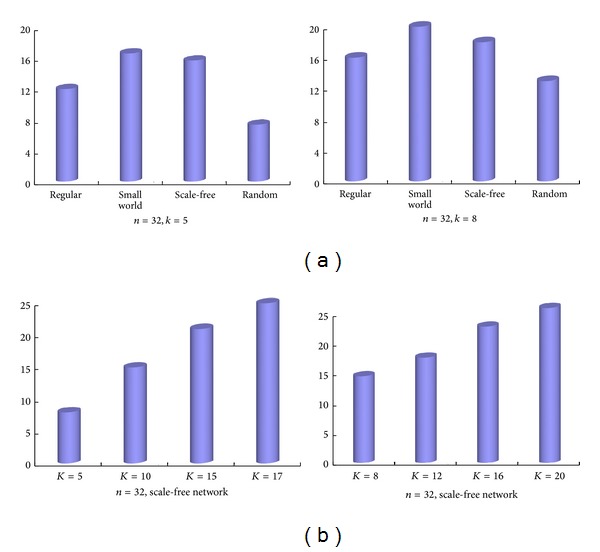
The effect of the network connection to the epileptic discharge propagation. (a) Given the network parameters (*n* = 32, *k* = 5 or 8), the propagation intensity of epileptic discharges on the regular, small world, scale-free, and random network. (b) The propagation intensity of epileptic discharges on the scale-free network of 32 nodes with the connectivity *K* = 5, 10, 15, and 17 or 8, 12, 16, and 20.

To investigate the effect of network connection to epileptic discharge propagation, we took the second test. One mass neural model (node) was adjusted to generate an epileptic discharge. Then the discharge of nodes (simulated EEG signals) was observed by changing the connection of network. Given the model parameters: *A* = 8 mV, *B* = 22 mV, and *K* ^*ij*^ = 400 and the network parameters *n* = 32, *k* = 5, the number of nodes that have an epileptic discharge was accounted for on the regular, small world, scale-free, or random networks, as shown in Figure 5(a). Particularly, as for the scale-free network, other parameters are maintained, just changing the connectivity *k* to 5, 10, 15, and 17, the number of nodes that had an epileptic discharge is accounted for and shown in Figure 5(b). Figure 5(a) shows that the small world and scale-free networks were the strongest to support the epileptic discharge propagation at the connectivity of 5 and 8. In the scale-free network, the epileptic discharge propagation increased linearly with the connectivity *k* (Figure 5(b)).

## 4. Conclusion

There are few studies concerning the mechanism of epileptic seizures on the complex network level. In this paper, a multiple mass neural model was used to construct different networks, including regular, random, small world, and scale-free networks. The simulation results showed that small world network and scale-free network are strongly correlated with the propagation of epileptic discharges. The findings suggested that the small world functional connectivity may have an intrinsic correlation with the synchronization of local neural networks, which act as a possible mechanism of epileptic seizure.
